# Improved production of biohydrogen in light-powered *Escherichia coli *by co-expression of proteorhodopsin and heterologous hydrogenase

**DOI:** 10.1186/1475-2859-11-2

**Published:** 2012-01-04

**Authors:** Jaoon YH Kim, Byung Hoon Jo, Younghwa Jo, Hyung Joon Cha

**Affiliations:** 1 Department of Chemical Engineering, Pohang University of Science and Technology, Pohang 790-784, Korea; 2 School of Interdisciplinary Bioscience and Bioengineering, Pohang University of Science and Technology, Pohang 790-784, Korea

**Keywords:** biohydrogen, *Escherichia coli*, proteorhodopsin, light-driven proton pump, light-powered cell factory

## Abstract

**Background:**

Solar energy is the ultimate energy source on the Earth. The conversion of solar energy into fuels and energy sources can be an ideal solution to address energy problems. The recent discovery of proteorhodopsin in uncultured marine γ-proteobacteria has made it possible to construct recombinant *Escherichia coli *with the function of light-driven proton pumps. Protons that translocate across membranes by proteorhodopsin generate a proton motive force for ATP synthesis by ATPase. Excess protons can also be substrates for hydrogen (H_2_) production by hydrogenase in the periplasmic space. In the present work, we investigated the effect of the co-expression of proteorhodopsin and hydrogenase on H_2 _production yield under light conditions.

**Results:**

Recombinant *E. coli *BL21(DE3) co-expressing proteorhodopsin and [NiFe]-hydrogenase from *Hydrogenovibrio marinus *produced ~1.3-fold more H_2 _in the presence of exogenous retinal than in the absence of retinal under light conditions (70 μmole photon/(m^2^·s)). We also observed the synergistic effect of proteorhodopsin with endogenous retinal on H_2 _production (~1.3-fold more) with a dual plasmid system compared to the strain with a single plasmid for the sole expression of hydrogenase. The increase of light intensity from 70 to 130 μmole photon/(m^2^·s) led to an increase (~1.8-fold) in H_2 _production from 287.3 to 525.7 mL H_2_/L-culture in the culture of recombinant *E. coli *co-expressing hydrogenase and proteorhodopsin in conjunction with endogenous retinal. The conversion efficiency of light energy to H_2 _achieved in this study was ~3.4%.

**Conclusion:**

Here, we report for the first time the potential application of proteorhodopsin for the production of biohydrogen, a promising alternative fuel. We showed that H_2 _production was enhanced by the co-expression of proteorhodopsin and [NiFe]-hydrogenase in recombinant *E. coli *BL21(DE3) in a light intensity-dependent manner. These results demonstrate that *E. coli *can be applied as light-powered cell factories for biohydrogen production by introducing proteorhodopsin.

## Background

Since the Industrial Revolution, energy consumption has increased exponentially and most energy has been derived from fossil fuels. Currently, we still depend on fossil fuels for more than 80 percent of our demands for electricity, transportation, and industries, although concerns about the exhaustion of fossil fuels and global warming have led to increased attention to renewable energy [1]. Among various renewable energy sources, solar energy is the most abundant and ultimate source. The total amount of solar energy absorbed by the Earth's surface is 1.74 × 10^5 ^terawatts (TW) [2], which is a tremendous amount compared to the world's energy consumption (~13 TW) [1]. Thus, the conversion of solar energy to fuels may constitue the most sustainable way to solve the energy crisis.

In the field of biotechnology, the photosynthetic process in algae and cyanobacteria has been actively investigated for the conversion of solar energy to useful biofuels [3-5]. Photosynthesis requires a highly complex photosystem composed of numerous proteins and photosynthetic enzymes, such as Rubisco [1]. In addition, many challenges still remain for engineering photosynthetic microorganisms [1,6]. Recently, a new type of rhodopsin, called proteorhodopsin, was discovered in the metagenome of uncultured marine γ-proteobacteria [7]. Proteorhodopsin can be heterologously expressed in *Escherichia coli *to possess proton-pumping activity [7], which is different from bacteriorhodopsin found in halobacteria [1,8]. This property of proteorhodopsin enables the investigation of its impact on cellular energy and phototrophy [8]. There have also been reports of the enhancement of cell viability or growth via light-driven proton pumping by proteorhodopsin under nutrient-limited conditions [9-11]. However, there have been no substantial applications in biofuel production using proteorhodopsin, although this potential has been mentioned recently [1].

Hydrogen (H_2_) has been recognized as one promising alternative energy source to fossil fuels. It does not emit carbon dioxide during combustion and can be easily converted to electricity using fuel cells. In addition, it has a higher energy density than other energy sources. Although the current production of H_2 _mainly depends on thermochemical methods using fossil fuels [12], biological approaches have been actively investigated to generate H_2 _in a more sustainable manner [13-17]. Among them, photobiological H_2 _production has attracted great attention due to its eco-friendly properties, such as its usage of solar energy and carbon assimilation. Nevertheless, there are still many obstacles to overcome, including slow cell growth, the low conversion efficiency of light to H_2_, the inhibitory effect of oxygen on hydrogenase activity, and others [16,17].

*E. coli *has been used widely as a cell factory for many types of bio-products (including biofuels), but it cannot utilize light energy. Therefore, constructing *E. coli *capable of absorbing light energy and converting it to other biofuels through the introduction of proteorhodopsin might increase biofuel production efficiency. It has been shown that protons generated by rhodopsin can migrate along the membrane surface [18] and thus, they can act as substrates of hydrogenase for H_2 _evolution. Thus, in the present work, for the first time (to our knowledge), we introduced proteorhodopsin into *E. coli*, generating bacteria capable of utilizing light and investigated its effect on H_2 _production yield using the previously constructed recombinant *E. coli *expressing *Hydrogenovibrio marinus*-originated [NiFe]-hydrogenase [15].

## Results

### Functional expression of proteorhodopsin in *E. coli*

**Figure 1:**
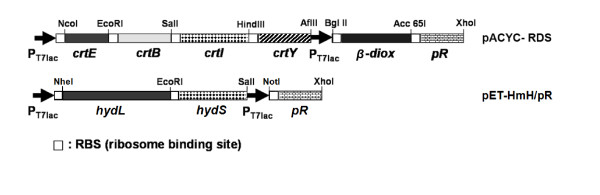
**Plasmid maps for the expression of proteorhodopsin in *E. coli***. *Erwinia uredovora crt E, B, I, Y *(for β-carotene synthesis), mouse *β-diox *gene (for conversion of β-carotene to retinal), and *pR *gene coding proteorhodopsin were cloned into pACYC-Duet1 vector to construct pACYC-RDS. All of the genes were amplified using the primers in Table 1 and digested using restriction enzymes for cloning into pACYC-Duet1. pET-HmH/pR was constructed by cloning a single *pR *gene into pET-HmH, which expresses *H. marinus *[NiFe]-hydrogenase, to investigate the function of proteorhodopsin with exogenous retinal. pACYC-pR (without *crtE, B, I, Y, β-diox*) was also constructed to measure the absorption spectrum of *E. coli *with membranes expressing proteorhodopsin (Figure 2B).

**Figure 2:**
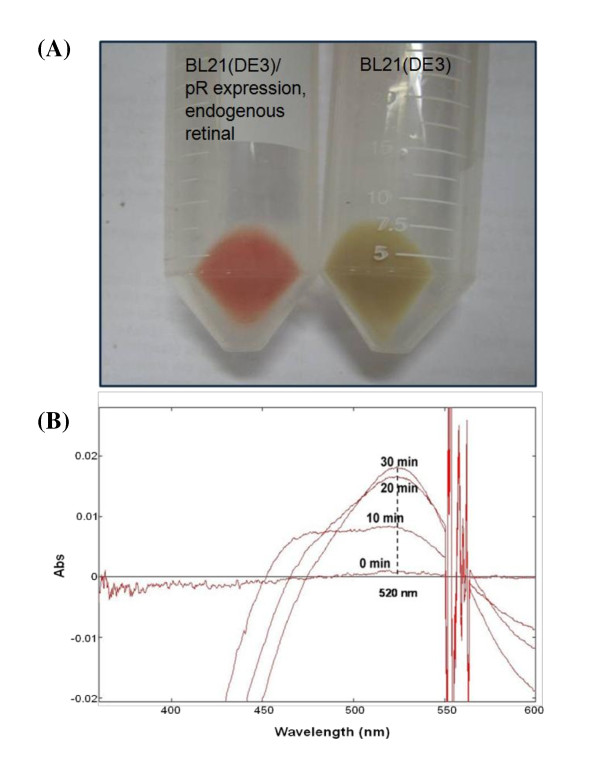
**Functional heterologous expression of proteorhodopsin in E. coli. (A)** Color-shift by expression of proteorhodopsin. Left sample: *E. coli *BL21(DE3)/pACYC-RDS (proteorhodopsin expression & retinal synthesis), right sample: wild-type *E. coli *BL21(DE3). **(B) Absorption spectra of the membrane of *E. coli *expressing proteorhodopsin alone**. Membrane fractions in 50 mM Tris-Cl (pH 8.0) and 5 mM MgCl_2 _were mixed with 20 μM all-trans-retinal. Spectra were measured every 10 min for 30 min.

*E. coli *does not have an intrinsic ability to absorb light energy. We constructed a plasmid, pACYC-RDS including 6 genes (*crtE, B, I, Y, b-diox*, and *pR*), that are required for the functional heterologous expression of proteorhodopsin in *E. coli *(Figure 1). The recombinant *E. coli *BL21(DE3) harboring pACYC-RDS was cultured, and protein expression was induced under exposure to 70 μmol photon/(m^2^·s) light. From the harvested cell pellet, we observed that the cells expressing proteorhodopsin with endogenous retinal have a distinctively reddish color compared to wild-type cells (Figure 2A). In addition, we confirmed that the membrane fraction, including recombinant proteorhodopsin (generated by the expression of a single *pR *gene), absorbs light at a specific wavelength of 520 nm in the presence of exogenous retinal, indicating the functional expression of recombinant proteorhodopsin in *E. coli *(Figure 2B).

### Co-expression effect of proteorhodopsin and hydrogenase on H_2 _production

**Figure 3:**
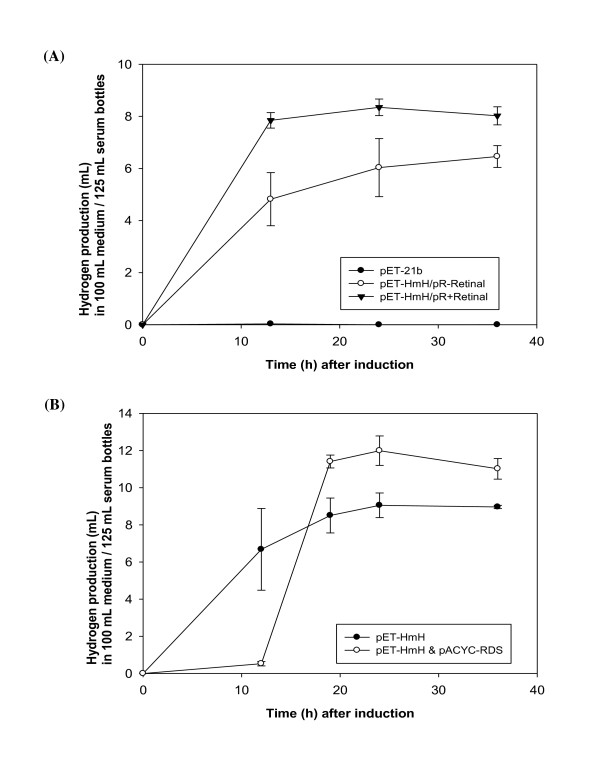
**Co-expression effect of proteorhodopsin and hydrogenase on H2 production. (A)** Co-expression effect of proteorhodopsin with exogenous retinal and hydrogenase on H_2 _production. Recombinant *E. coli *BL21(DE3) with a single plasmid, pET-HmH/pR, were grown with or without retinal under a light intensity of 70 μmole photon/(m^2^·s). *E. coli *harboring the parent pET-21b vector was used as a negative control. **(B) Co-expression effect of proteorhodopsin with endogenous retinal and hydrogenase on H_2 _production**. Recombinant *E. coli *BL21(DE3) with two plasmids, pET-HmH and pACYC-RDS, were grown under the same light conditions. H_2 _production was measured using GC as described in the Methods. Each value and its error bars represent the mean of two independent cultures and the standard deviation, respectively.

After confirmation of proteorhodopsin function in recombinant *E. coli*, we investigated the effect of co-expressing proteorhodopsin and *H. marinus *[NiFe]-hydrogenase on H_2 _production. We used two kinds of expression systems: a single plasmid system of pET-HmH/pR (without endogenous retinal) and a dual plasmid system of pET-HmH and pACYC-RDS (with endogenous retinal) (Figure 1). *E. coli *BL21(DE3) transformed with pET-HmH/pR or cotransformed with pET-HmH and pACYC-RDS was cultured in 125 mL serum bottles under exposure to 70 μmol photon/(m^2^·s) light. We found that *E. coli *with pET-HmH/pR produced more H_2 _after retinal addition under light than the cells without retinal (Figure 3A). This result indicates that the gained function of recombinant proteorhodopsin by the addition of retinal has a synergistic effect on H_2 _production with the heterologous expression of hydrogenase. A negative control strain containing the parent vector (pET-21b) did not produce H_2 _under the same conditions. Using the dual plasmid system for endogenous retinal biosynthesis, we also observed similar results (Figure 3B). The BL21(DE3) strain with the dual plasmid system (pET-HmH and pACYC-RDS) also produced ~1.3-fold more H_2 _compared to the strain expressing hydrogenase (pET-HmH) alone. Although the strain harboring two plasmids showed lower H_2 _production than the strain expressing only hydrogenase at 12 h, its production rapidly increased and surpassed the H_2 _production of the hydrogenase-only strain after 19 h (Figure 3B).

### Light intensity effect on H_2 _production by co-expression of proteorhodopsin and hydrogenase

**Figure 4:**
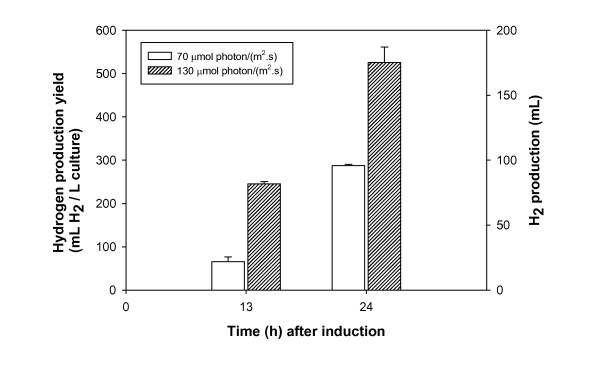
**The effect of light intensity on H_2 _production by co-expression of proteorhodopsin and hydrogenase**. Recombinant *E. coli *BL21(DE3) with two plasmids, pET-HmH and pACYC-RDS, were grown in 500 mL sealed serum bottles containing 350 mL M9 medium. Light intensity was adjusted to 70 μmole photon/(m^2^·s) or 130 μmole photon/(m^2^·s). H_2 _production was measured using GC. Each value and error bars represent the mean of two independent cultures and the standard deviation.

To investigate the effect of light energy on H_2 _production by the co-expression of proteorhodopsin and hydrogenase, light intensity was changed during the culture. We increased light intensity from 70 μmole photon/(m^2^·s) to 130 μmole photon/(m^2^·s) at the middle part of culture bottles by changing the light source from two 20 W fluorescent lamps to two 30 W fluorescent lamps. We observed that the cells cultured at a light intensity of 130 μmole photon/(m^2^·s) produced more H_2 _than those cultured at 70 μmole photon/(m^2^·s) (Figure 4). At 24 h after induction, the cells grown under 130 μmole/(m^2^·s) light produced 184 ± 8.9 mL H_2 _while cells grown under 70 μmole photon/(m^2^·s) light produced 100.5 ± 0.8 mL H_2_, corresponding to yields of 525.7 ± 25.4 mL H_2_/L-culture and 287.3 ± 2.1 mL H_2_/L-culture, respectively. A production rate of 21.9 mL H_2_/(L-culture·h) was achieved from the culture for 24 h at a light intensity of 130 μmole photon/(m^2^·s). Cell growth was similar between the two cultures under different light conditions (data not shown). This indicated that improved H_2 _production (0.0835 L H_2_, equivalent to 0.902 kJ) was derived from enhanced light energy (60 μmole photon/(m^2^·s) for 24 h, equivalent to 26.579 kJ). Thus, we might determine that the conversion efficiency of light energy to H_2 _was ~3.4% in the present work using recombinant *E. coli *BL21 expressing both proteorhodopsin and *H. marinus *[NiFe]-hydrogenase.

## Discussion

**Figure 5:**
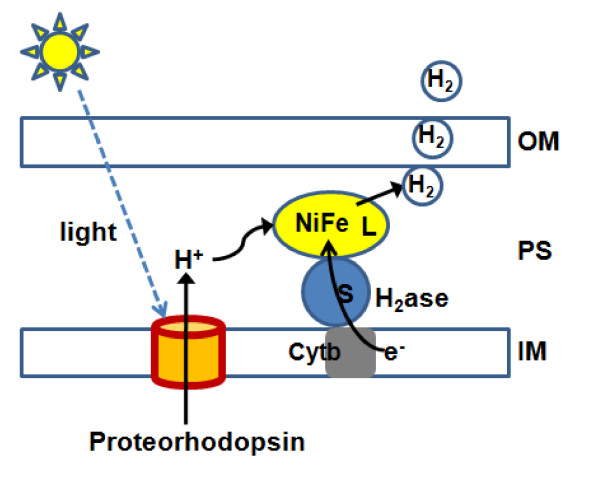
**Schematic diagram of H_2 _production by the co-expression of proteorhodopsin and [NiFe]-hydrogenase in *E. coli***. Proteorhodopsin transports protons across the membrane by absorption of light energy. Protons are transferred to the active site in the large subunit (L) of [NiFe]-hydrogenase in the periplasm and reduced to H_2 _by the addition of an electron, which is transferred thorough [Fe-S] clusters in the small subunit of [NiFe]-hydrogenase (S) and *b*-type cytochrome (Cyt b, encoded by *hyaC *in *E. coli*) in the inner membrane. Abbreviations: OM, outer membrane; PS, periplasmic space; IM, inner membrane.

*E. coli *is a chemotroph that cannot utilize light energy. Bacteriorhodopsins, including proteorhodopsin, are the simplest molecules that enable microorganisms to utilize solar energy to create a proton gradient and generate ATP. In addition, protons translocated by rhodopsin can migrate along the membrane [18] and might be substrates of hydrogenase for the evolution of H_2 _(Figure 5). Thus, we tried to convert light energy to H_2 _by exploiting the light-driven proton-pumping function of proteorhodopsin in recombinant *E. coli *expressing hydrogenase.

We observed functional expression of proteorhodopsin in the recombinant *E. coli *BL21(DE3) and thus investigated the effect of co-expressing proteorhodopsin and *H. marinus *[NiFe]-hydrogenase on H_2 _production. Using a single plasmid system expressing both hydrogenase and proteorhodopsin without biosynthesis of endogenous retinal, we found that H_2 _production was improved by ~29% in the presence of added retinal under light. This indicates that proteorhodopsin function, gained with exogenous retinal, provided a synergistic effect on H_2 _production through the supplementation of protons. Similarly to the single plasmid system, we also observed a positive effect of proteorhodopsin using a dual plasmid system. H_2 _production levels of cells co-expressing hydrogenase, proteorhodopsin, and retinal synthesis proteins was ~1.3-fold higher at the final time point than that of the cells expressing only hydrogenase. It is noteworthy that H_2 _production from the strain co-expressing proteorhodopsin and hydrogenase quickly surpassed H_2 _production from the strain expressing only hydrogenase at a late phase. This retarded H_2 _production profile of the dual plasmid system might be attributed to the metabolic burden caused by the over-expression of multiple proteins required for the biosynthesis of retinal and proteorhodopsin.

Because the function of proteorhodopsin is driven by the absorption of light energy, light intensity can be a key factor for H_2 _production. We found that the H_2 _production from cells co-expressing hydrogenase and proteorhodopsin with endogenous retinal is strongly dependent on light intensity. Increasing the light intensity from 70 μmole photon/(m^2^·s) to 130 μmole photon/(m^2^·s) increased H_2 _production yield ~1.8-fold. However, cell growth was not different in the two cultures under different light conditions. This tendency was consistent with a previous report that proteorhodopsin contributes to cell growth only under nutrient-limited conditions [9]. Thus, this indicates that H_2 _production was improved by the absorption of enhanced light energy through the proton-pumping function of proteorhodopsin. In the present study, it seems that the reduction from proton to H_2 _mediated by hydrogenase generates additional proton gradient, driving proteorhodopsin to pump protons, the substrates for hydrogenase. When we calculated the production rate per culture volume (21.9 mL H_2_/(L-culture·h)) and the conversion efficiency of light energy to H_2 _(~3.4%), the levels achieved in this study were comparable to the results of photobiological hydrogen production in previous studies: H_2 _production rate in green algae, 0.048-4.48 mL H_2_/(L-culture·h), cyanobacteria, 4.03-13 mL H_2_/(L-culture·h), photosynthetic bacteria, 7.6-131 mL H_2_/(L-culture·h), and light conversion efficiency in photoautotrophs, 3-10% with removal of O_2 _or 1-2%, and photoheterotrophs, 0.308-9.23%) [17,19]. Although cell growth was mainly supported by exogenous nutrients in this system, these results are quite meaningful for the development of an *E. coli *system that can utilize light energy as a supplementary source.

## Conclusions

Here, we demonstrated the substantial application of proteorhodopsin for light-driven biohydrogen production in a recombinant *E. coli *system. *E. coli *engineered to express *H. marinus *[NiFe]-hydrogenase and proteorhodopsin produced more H_2 _with the existence of retinal under light conditions. Engineered strains also produced more H_2 _as light intensity increased, although there was no difference in cell growth. These results suggest that our system works for converting light energy to H_2 _via the cooperation of proteorhodopsin and hydrogenase. In addition, engineering *E. coli *as light-powered cell factories could provide a solution for developing potential strategies for photobiological H_2 _production.

## Methods

### Bacterial strains and culture conditions

*E. coli Top10 *(Invitrogen, USA) was used for the manipulation and cloning of target genes. *E. coli *BL21(DE3) (Novagen, USA) was used for expression of recombinant proteins. We used LB medium including proper antibiotics (50 μg/mL ampicillin and 30 μg/mL chloramphenicol) for genetic manipulation. For protein expression, 1 mM (as a final concentration) of isopropyl-β-D-thiogalactopyranoside (IPTG; BioBasic, Canada) was added to each culture medium. For *in vivo *H_2 _production, cells were grown in M9 minimal medium (6 g/L Na_2_HPO_4_, 3 g/L KH_2_PO_4_, 1 g/L NH_4_Cl, 0.5 g/L NaCl, and 1 mg/L thiamine supplemented with 240.73 mg/L MgSO_4_, 11.09 mg/L CaCl_2_) including 5 g/L casamino acids and 5 g/L glucose. 0.1 M of FeSO_4 _and 1 M of NiSO_4 _solutions were added to M9 medium at a concentration of 30 μM for the functional expression of *H. marinus *[NiFe]-hydrogenase. All cultures were maintained under normal aerobic or micro-aerobic conditions [14]. Cells were grown in serum bottles (125 or 500 mL; Wheaton, USA) sealed with rubber stoppers and aluminum capping at 37°C in an air-shaking incubator (Jeiotech, Korea) at a gyration rate of 200-230 rpm. For the assessment of the functional activity of proteorhodopsin, cells were irradiated by 20 W or 30 W fluorescent lamps. The light intensity (400-700 nm) at a given location in the culture was measured using a light meter (Apogee, USA) in units of μmol photon/(m^2^·s).

### Plasmid construction

For the expression of proteorhodopsin in *E. coli *BL21(DE3), four genes (*Erwinia uredovora crt E, B, I, Y*; Genbank: D90087) for β-carotene synthesis and *β-diox *gene for the conversion of β-carotene to retinal (mouse β-carotene-15,15'-dioxygenase; Genbank: AF271298) were obtained from pORANGE and β-plasmid (a kind gift from Dr. J. von Lintig), respectively [20]. *pR *gene coding for proteorhodopsin (Genbank: AF279106) was also obtained from BAC clone EBAC31A08 (a kind gift from Dr. E. Delong) [7]. All six genes above were amplified by polymerase chain reaction (PCR) using the primers in Table 1 and cloned into the pACYCDuet-1 (Novagen) vector to construct pACYC-RDS (Figure 1), which is compatible with the pET vector. For the expression of *H. marinus *[NiFe]-hydrogenase genes, the pET-HmH vector was used [15]. To analyze the effect of functional proteorhodopsin with retinal, the proteorhodopsin gene (*pR*), without the genes for retinal synthesis, was cloned into the pET-HmH vector to construct pET-HmH/pR (Figure 1).

**Table 1:** Primer sequences for amplification of genes related to β-carotene synthesis, retinal synthesis, and proteorhodopsin

Primer name	Sequence (5'→3') (___: ribosome binding site,:___restriction site)
*crtE *forward	GCCCATGGATGACGGTCTGCGCAAAAAAACACG

*crtE *reverse	GCGAATTCTTAACTGACGGCAGCGAGTTTTTTG

*crtB *forward	CCGAATTCAAGGAGATATACCAATGAATAATCCGTCGTTACT CAATCATGC

*crtB *reverse	CGGTCGACCTAGAGCGGGCGCTGCCAGAG

*crtI *forward	CCGTCGACAAGGAGATATACAAATGAAACCAACTACGGTAAT TG

*crtI *reverse	CGAAGCTTTCATATCAGATCCTCCAGCATC

*crtY *forward	CCAAGCTTGAAGGAGATATACCAATGCAACCGCACTATGATC TGATTCTC

*crtY *reverse	GCCTTAAGTTAGCGATGAGTCGTCATAATGGC

*β-diox *forward	GGAGATCTAAGGAGATATACATATGGAGATAATATTTGGCCA GAATAAG

*β-diox *reverse	CCGGTACCTTAAAGACTTGAGCCACCATGACCC

*pR *forward	CCGGTACCAAGGAGATATACAAATGGGTAAATTATTACTGAT ATTAGGTAG CCGCGGCCGCAAGGAGATATACAAATGGGTAAATTATTACTGAT ATTAGGTAG

*pR *reverse	GCCTCGAGTTAAGCATTAGAAGATTCTTTAACAGCAAC

### Measurement of *In vivo *H_2 _production

H_2 _gas produced in cell culture was obtained from the headspace of a sealed serum bottle (125 or 500 mL). Usually, 20-100 μL of the gas sample was analyzed using a gas chromatograph (GC; Younglin Instrument, Korea) equipped with a carboxen-1010 PLOT column (0.53 mm × 30 m, Supelco, USA) and pulsed discharge detector (Valco Instrument, USA). Elution was performed using helium as a carrier gas at a flow rate of 10 mL/min, and the temperatures of the injector, detector, and oven were set to 130, 250, and 100°C, respectively. The H_2 _concentration in the gas sample was calculated using a standard curve. The H_2 _amount was determined based on the H_2 _concentration and gas volume of headspace (including expanded volume).

### Measurement of absorption spectra of proteorhodopsin

The absorption spectrum of cells expressing proteorhodopsin was measured using spectrophotometry [7]. To prepare crude membrane fractions, recombinant *E. coli *were harvested by centrifugation at 4°C and 4,000 rpm for 15 min. The cell pellet was resuspended in 50 mM Tris-Cl (pH 6.8) and disrupted with a sonic dismembrator (Fisher Scientific, USA) for 10 min at 50% power (5 sec pulse on and 2 sec pulse off). The disrupted cell suspension (total cell lysate) was centrifuged at 4°C and 10,000 *g *for 20 min. The resultant supernatant (crude extract) was centrifuged at 4°C and 120,000 *g *for 120 min. The pellet was resuspended in 50 mM Tris-Cl (pH 8.0) and 5 mM MgCl_2_, which is regarded as the crude membrane. The absorption spectrum was measured at spectrum mode using a spectrophotometer (Shimadzu, Japan) after the addition of 20 μM of all-trans-retinal (Sigma).

### Conversion efficiency of light energy to H_2_

The conversion efficiency of light energy to H_2 _was calculated using the following equation: conversion efficiency (%) = (H_2 _production amount × H_2 _energy content)/absorbed light energy × 100, where H_2 _energy content = 10.8 kJ/L (lower heating value of H_2_) [19]. Absorbed light energy was calculated using the equation absorbed light energy (kJ/s) = 0.2176 I_o_S_A_, where I_o _= incident light intensity (μmol/(m^2^·s) measured by light meter, and S_A _= illuminated surface area (m^2^) = π × d × h (d = 0.075 m, h = 0.1 m for the culture in a 500 mL serum bottle) [21].

## Competing interests

The authors declare that they have no competing interests.

## Authors' contributions

JYHK and HJC designed research. JYHK, BHJ, and YJ performed and analyzed biohydrogen production in recombinant *E. coli*. JYHK and HJC wrote the paper. All authors have read and approved the final version of the manuscript.
